# Genetic structure of South African Nguni (Zulu) sheep populations reveals admixture with exotic breeds

**DOI:** 10.1371/journal.pone.0196276

**Published:** 2018-04-26

**Authors:** Mokhethi Matthews Selepe, Simone Ceccobelli, Emiliano Lasagna, Nokuthula Winfred Kunene

**Affiliations:** 1 Department of Agriculture, University of Zululand, Kwadlangezwa, South Africa; 2 Department of Agricultural, Food and Environmental Sciences, University of Perugia, Perugia, Italy; National Cheng Kung University, TAIWAN

## Abstract

The population of Zulu sheep is reported to have declined by 7.4% between 2007 and 2011 due to crossbreeding. There is insufficient information on the genetic diversity of the Zulu sheep populations in the different area of KwaZulu Natal where they are reared. The study investigated genetic variation and genetic structure within and among eight Zulu sheep populations using 26 microsatellite markers. In addition, Damara, Dorper and South African Merino breeds were included to assess the genetic relationship between these breeds and the Zulu sheep. The results showed that there is considerable genetic diversity among the Zulu sheep populations (expected heterozygosity ranging from 0.57 to 0.69) and the level of inbreeding was not remarkable. The structure analysis results revealed that Makhathini Research Station and UNIZULU research station share common genetic structure, while three populations (Nongoma, Ulundi and Nquthu) had some admixture with the exotic Dorper breed. Thus, there is a need for sustainable breeding and conservation programmes to control the gene flow, in order to stop possible genetic dilution of the Zulu sheep.

## Introduction

Human history has been completely transformed by the domestication of animals and plants over the past 10,000 years [1]. Their domestication had a critical influence on demographic trends and was a requirement for the rise and development of civilisation [2, 3]. Archaeozoologists have stated that the domestication of animals according to the criterion of body size reduction is said to have begun with goats and then sheep about 10,000 to 9,500 B.P ago, approximately 1,000 years after plant domestication [4]. The domestic stocks in Southern Africa were initially acquired by nomadic people (KhoiKhoi) in Botswana around 2300 BP. The main southward dispersal route of animals to Southern Africa was through the Kalahari desert, the Orange River, or either the route that runs parallel to the western coast of Namibia [5]. The KhoiKhoi people moved their livestock around searching for enough grazing for them and they migrated according to the season [6].

Sheep play an essential role in the livelihood of people around the world as they are a source of meat, milk, wool, hide and manure, especially in developing countries [7] [8] [9]. In South Africa, wool production plays an important economic role as an earner of foreign exchange. This is because as an export product, more than 90% of the total production is exported either as greasy wool or in semi-processed form as scoured and wool top. South African wool production is mainly Merino and Karakul, but coarse and coloured types are also produced and marketed on a limited scale [10]. In particular, local breeds can be considered as reservoirs of genetic diversity. Indeed, they have evolved over centuries managed by traditional pastoralists, enabling the emergence of a wide diversity [11], of strong adaptations to harsh conditions, nutritional fluctuations, and resistance to diseases and parasites [12] [13] [14]. Human socio-cultural and economic networks have had a marked impact on their genetic makeup.

Zulu sheep are one of four ecotypes of the Nguni sheep breed (Landim, Pedi, Swazi and Zulu) [15] [16]. The Nguni people brought the ancestors of this breed to the east coast of South Africa (KwaZulu Natal) between 200 and 400 AD [17]. In their southward migration, Nguni people migrated along the eastern part of Southern Africa from Central and East Africa during the 16^th^ and 17^th^ centuries. Some of the migrants settled along the way, while the other group travelled further. This gave rise to the division of Nguni people. The Zulu people, who took their name from their first ruler or king, ''Shaka Zulu'', settled in the green plains of KwaZulu Natal [18]. The farmers in the rural communities of KwaZulu Natal keep Zulu sheep as a source of protein and for sale [19]. This Nguni sheep ecotype is characterised by having either thin or fat tail (carrot shaped), multicolours, and a coat of either wool or hair [15]. Moreover, the Zulu sheep have acquired high adaptation to harsh environmental conditions, resistance against diseases and parasites and, the ability to walk long distances. The Zulu sheep can be distinguished from other Nguni breeds by their small mouse ears, and they appear to be more woolly. In addition, the dominant colours are brown and white, black and brown, and a unique fawn colour [16] [19]. However, the population of Zulu sheep is reported to be declining, due to cross-breeding with exotic breeds, especially with the Dorper and Merino sheep breeds [16] [20] [21] with the aim of increasing body weight [16]. Uncontrolled mating strategies may result in genetic erosion of Zulu sheep, leading to their eventual extinction [22].

There is currently a gap in information available on the genetic variation among Zulu sheep populations. The research published so far by Kunene et al. [20] and Hlophe [23] on genetic characterisation covered a few populations of Zulu sheep. In these studies, the authors concluded that there is a notable level of inbreeding among Zulu sheep and thus a ram exchange programme should be implemented. Furthermore, the authors recommended that more Zulu sheep populations should be analysed in comparison with Dorper, Damara and Merino, since crossbreeding was suspected in some areas. According to Mavule et al. [21] the spread of Zulu sheep into different areas of KwaZulu Natal has fractured the sheep into isolated subpopulations occupying different ecological, social-cultural and management environments. However, this study was based on morphological features, thus assessment at molecular level is required.

The aims of this study were two-fold: (i) to confirm or disconfirm the structuration among eight Zulu sheep populations revealed by morphological analysis, using molecular tools; (ii) to assess the phenomenon of crossbreeding, in particular with Dorper and Merino sheep, using microsatellites. Microsatellites are characterised as co-dominant inheritance, highly distributed throughout the genome, showing a high mutation rate and a high level of polymorphism [24] [25] [26]. They are one of widely used markers to assess genetic variation, genetic relationship, and population structure of sheep breeds [27] [28] [29] [30] [31]. The microsatellite markers are still useful to assess genetic structure of sheep although there are new other recommended techniques. Nevertheless, there are a lot of advantages in using new generation molecular markers such as single nucleotide polymorphisms (SNPs) as already reported in recent literature [32]. The newly generated genome-wide data are in fact valuable resources for the future conservation and genetic improvement of domestic sheep and can also serve as a valuable resource for genomics-assisted breeding [33].

The molecular information obtained in the present study will serve as a guideline for management and breeding strategies (reducing inbreeding and crossbreeding) for better utilisation and conservation of Zulu sheep; this is recommended to avoid unscientific conservation decisions made at political level [34].

## Results

### Genetic variation

In total, 323 alleles were detected across the 28 microsatellites loci in the studied Zulu sheep populations and three exotic breeds with a mean of 11.54 alleles per locus (S1 Table). The most polymorphic marker with the highest number of alleles per locus was HSC (19), whereas ETH10 had the lowest number of alleles per locus (3). The polymorphic information content (PIC) per locus ranged from 0.11 (ETH10) to 0.80 (HSC) (S1 Table). PIC values revealed that all markers were informative with the exception of ETH10 which was thus excluded for further statistical analysis. As significant deviation from Hardy-Weinberg equilibrium was detected in the populations studied, the locus TGLA126 was also excluded for further statistical analysis. The results of the genetic diversity, genetic distance and breed assignment are based on the 26 remaining microsatellite markers.

The mean number of observed alleles (MNA) ranged from 3.84 (UZ) to 6.64 (NQ) (Table 1). After adopting the rarefaction methodology, the mean allelic richness ranged from 3.53 (ES) to 6.29 (NQ) in a sample size of 12 individuals. Distribution of allelic richness and distribution of private allelic richness were significantly different (respectively: Kruskal-Wallis chi-squared = 42.15, df = 10, p-value = <0.0001; Kruskal-Wallis chi-squared = 38.10, df = 10, p-value = <0.0001) between populations.

**Table 1:** Genetic diversity of the studied sheep breeds obtained from the analysis of 26 microsatellite loci.

Breed /population	ID	Sample Size	MNA ± SD	R(PR)	HO ± SD	HE ± SD	*F*_IS_ [IC_95%_]
Jozini	JO	30	5.69±1.87	3.94 (0.11)	0.53±0.02	0.63±0.03	0.16 [0.08–0.20]
Mtubatuba	MT	29	5.58±1.77	3.91 (0.16)	0.57±0.02	0.62±0.04	0.09 [-0.00–0.13]
Nongoma	NG	30	5.85±1.59	4.05 (0.19)	0.56±0.02	0.65±0.03	0.13 [0.04–0.17]
Eshowe	ES	19	3.73±1.34	3.14 (0.04)	0.58±0.02	0.57±0.03	-0.01 [-0.10–0.02]
Ulundi	UL	23	5.54±1.70	4.04 (0.24)	0.59±0.02	0.66±0.03	0.11 [-0.00–0.16]
Nquthu	NQ	22	6.50±1.75	4.44 (0.25)	0.62±0.02	0.69±0.02	0.12 [0.05–0.13]
UNIZULU research station^1^	UZ	21	4.00±1.57	3.35 (0.14)	0.58±0.02	0.60±0.03	0.03 [-0.09–0.09]
Makhathini research station^1^	MS	33	5.46±2.47	3.83 (0.29)	0.60±0.02	0.64±0.03	0.06 [-0.02–0.12]
Dorper	DO	23	4.92±1.57	3.69 (0.17)	0.57±0.02	0.61±0.03	0.06 [-0.04–0.11]
Damara	DA	29	6.12±1.73	4.24 (0.29)	0.63±0.02	0.67±0.04	0.07 [0.01–0.09]
South African Merino	ME	30	6.08±1.92	4.31 (0.38)	0.67±0.02	0.70±0.03	0.04 [-0.01–0.06]

ID: population/breed acronyms, N: sample size of each breed, MNA: mean number of observed alleles, R: allelic richness, PR: private allelic richness, H_O_: mean observed heterozygosity, H_E:_ expected heterozygosity, *F*_IS_: inbreeding coefficient per breed.

^1^Data taken from Kunene et al. [20].

The highest observed heterozygosity (H_O_) was detected in ME (0.67), while JO showed the lowest (0.53). The highest expected heterozygosity (H_E_) (with the exception of the exotic breeds) was observed in NQ (0.69), with the lowest (0.57) in ES (Table 1). Distribution of H_O_ was not significantly different between populations (Kruskal-Wallis chi-squared = 13.80, df = 10, p-value = 0.18), whereas distribution of H_E_ was significantly different between populations (Kruskal-Wallis chi-squared = 20.93, df = 10, p-value = 0.02).

The inbreeding coefficient (*F*_IS_) estimated ranged from -0.01 (ES) to 0.16 (JO) (Table 1).

The two-phase mutation model under Wilcoxon sign rank tests was utilised to find out recent bottlenecks (heterozygosity excess) in the Zulu sheep populations. The heterozygosity excess obtained (data not shown) were significant (*P*<0.05) in ES (0.005), UZ (0.003) and MS (0.03).

### Genetic differentiation, distance and phylogeny

Pairwise genetic differentiation (*F*_ST_) among populations is shown in Table 2. The *F*_ST_ genetic distance estimate values revealed that the closest populations were NQ and NG (0.056) and MS and UZ (0.070), while the longest distance (0.235) between the Zulu sheep populations was realised between ES and MS. DO was more genetically distant from UZ and MS (0.272 and 0.255, respectively) than to the other Zulu sheep populations. The NQ population was genetically the closest population to the DO sheep breed.

**Table 2:** Pairwise *F*_ST_ among the studied breeds/populations (with confidence intervals at 95%).

	JO	MT	NG	ES	UL	NQ	UZ	MS	DO	DA
**MT**	0.110 [0.050–0.181]									
**NG**	0.082 [0.046–0.122]	0.142 [0.082–0.208]								
**ES**	0.159 [0.079–0.253]	0.209 [0.124–0.293]	0.095 [0.067–0.132]							
**UL**	0.146 [0.087–0.220]	0.180 [0.103–0.255]	0.072 [0.040–0.110]	0.101 [0.071–0.133]						
**NQ**	0.093 [0.055–0.141]	0.142 [0.073–0.209]	0.056 [0.037–0.077]	0.118 [0.069–0.185]	0.081 [0.044–0.134]					
**UZ**	0.204 [0.134–0.285]	0.184 [0.109–0.273]	0.219 [0.153–0.293]	0.228 [0.128–0.331]	0.233 [0.162–0.301]	0.226 [0.162–0.294]				
**MS**	0.158 [0.093–0.234]	0.150 [0.085–0.228]	0.202 [0.143–0.266]	0.235 [0.154–0.316]	0.220 [0.146–0.294]	0.207 [0.141–0.273]	0.070 [0.038–0.106]			
**DO**	0.131 [0.085–0.177]	0.205 [0.136–0.284]	0.111 [0.077–0.146]	0.143 [0.105–0.182]	0.132 [0.103–0.162]	0.084 [0.060–0.110]	0.272 [0.206–0.343]	0.255 [0.187–0.326]		
**DA**	0.144 [0.098–0.201]	0.132 [0.092–0.179]	0.153 [0.110–0.196]	0.203 [0.137–0.273]	0.168 [0.110–0.243]	0.167 [0.113–0.231]	0.147 [0.095–0.212]	0.110 [0.069–0.157]	0.220 [0.152–0.291]	
**ME**	0.161 [0.119–0.205]	0.159 [0.128–0.195]	0.170 [0.128–0.212]	0.215 [0.163–0.268]	0.173 [0.115–0.246]	0.162 [0.115–0.214]	0.185 [0.139–0.238]	0.155 [0.121–0.190]	0.216 [0.162–0.269]	0.090 [0.061–0.118]

JO, Jozini; MT, Mtubatuba; NG, Nongoma; ES, Eshowe; UL, Ulundi; NQ, Nquthu; UZ, UNIZULU research station; MS, Makhathini research station; DO, Dorper; DA, Damara; ME, South African Merino.

**Fig 1:**
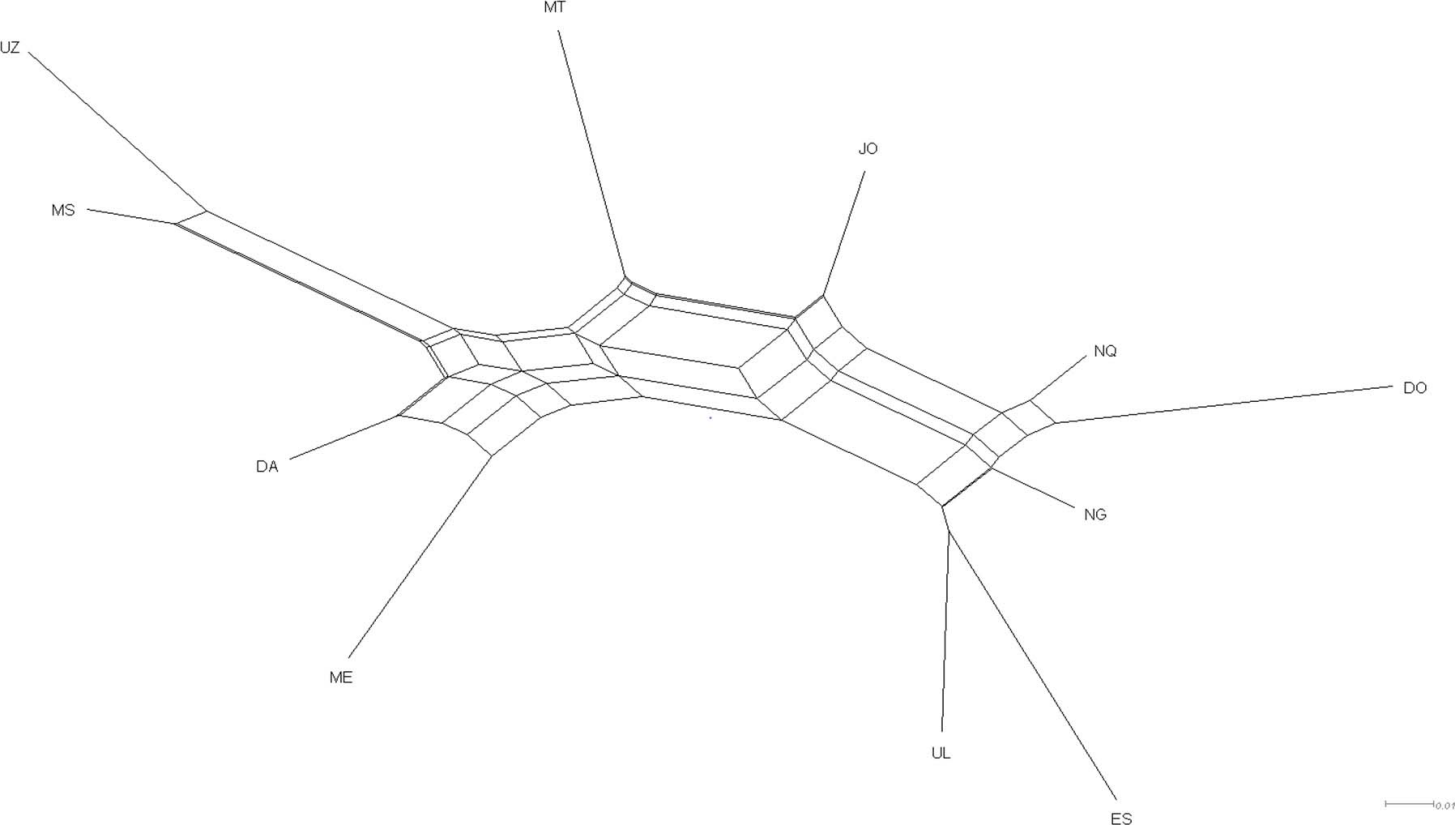
Genetic relationship among the 11 sheep populations using reynolds genetic distance according to the neighbour-joining algorithm. JO, Jozini; MT, Mtubatuba; NG, Nongoma; ES, Eshowe; UL, Ulundi; NQ, Nquthu; UZ, UNIZULU research station; MS, Makhathini research station; DO, Dorper; DA, Damara; ME, South African Merino.

The neighbor-joining tree obtained from Reynolds weighted genetic distance (Fig 1) showed the genetic relationship among 11 sheep populations. The phylogenetic tree revealed two clear clusters, the first cluster comprising MS and UZ, with the second comprising NQ, DO, UL, ES and NG. The remaining 4 populations (JO, MT, DA and ME) could not be grouped in any cluster.

### Genetic structure and admixture analysis

**Fig 2:**
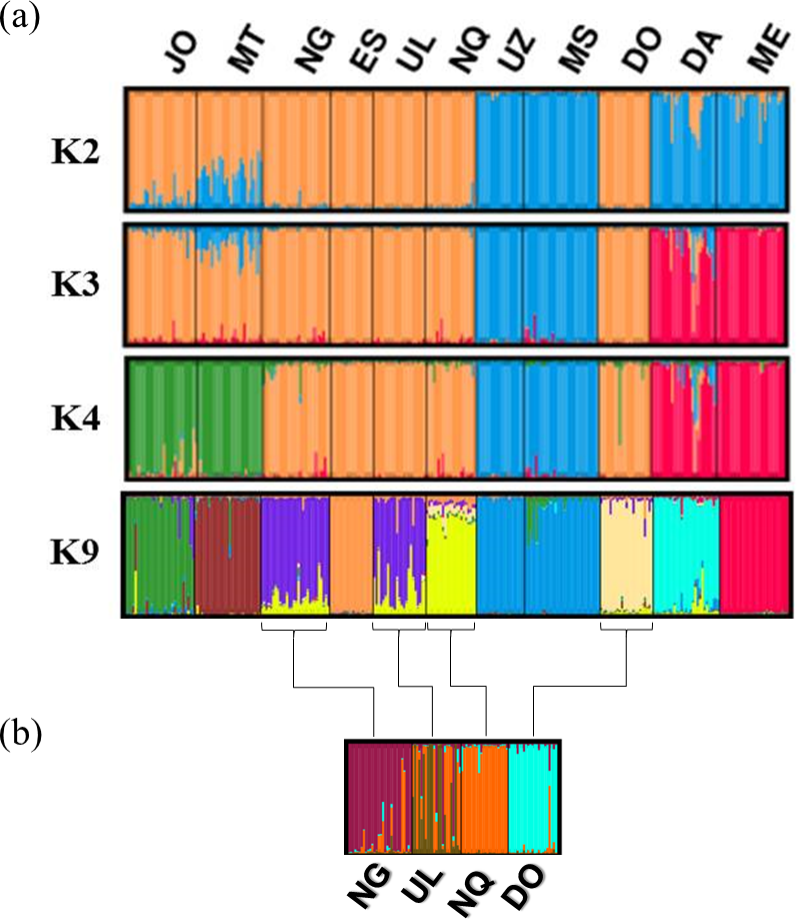
Genetic clustering of 11 sheep population with STRUCTURE. **(a) Analysis of the entire data set obtained from 10 runs for each number of assumed populations (*K*) value ranging from 2 to 9; (b) further analysis obtained from four populations (NG, UL, NQ and DO).**JO, Jozini; MT, Mtubatuba; NG, Nongoma; ES, Eshowe; UL, Ulundi; NQ, Nquthu; UZ, UNIZULU research station; MS, Makhathini research station; DO, Dorper; DA, Damara; ME, South African Merino.

**Fig 3:**
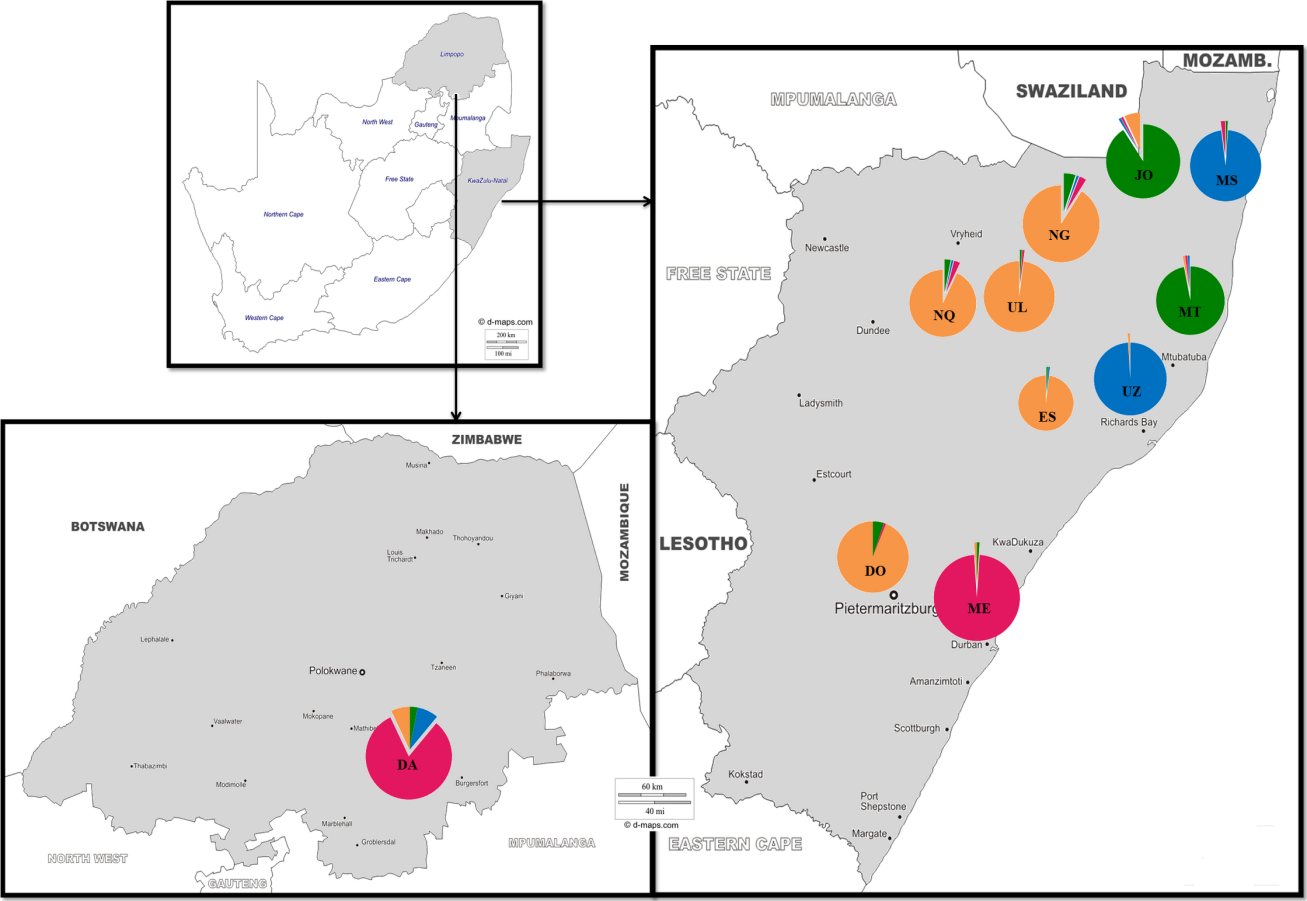
Representation of sample size (indicated by diameter of the pies) and of relative admixture distribution (indicated by different colour) inferred by STRUCTURE at *K* = 4. JO, Jozini; MT, Mtubatuba; NG, Nongoma; ES, Eshowe; UL, Ulundi; NQ, Nquthu; UZ, UNIZULU research station; MS, Makhathini research station; DO, Dorper; DA, Damara; ME, South African Merino.Source: figure taken from http://www.d-maps.com and adapted for illustrative purpose only.

The populations’ structure (Fig 2) was analysed using Bayesian clustering analysis to determine the number of clusters (*K*) present in the populations, permitting the identification of differences among populations and hidden substructures within them. The highest Δ*K* value was detected at *K* = 4 (S2A Fig). The Q-matrix averaged over the most similar run for *K* = 4, was used to display in a map the distribution of membership coefficients according to population and geographical location (Fig 3). As shown in Fig 2, at *K* = 2, two clusters were formed; UZ, MS, DA and ME clustered together, while JO, NG, ES, UL, NQ and DO formed a second cluster. MT appeared as an admixture between the two clusters. At *K* = 3, DA and ME clustered together separately. At *K* = 4, four different clusters were defined; the first cluster (JO and MT), the second cluster (NG, ES, UL, NQ and DO), the third cluster (UZ and MS) and the fourth cluster (DA and ME). However, DA and ME contained some individuals associated with other eight Zulu sheep populations (not well differentiated), while DO related to NG, ES, UL and NQ. The lower Δ*K* peak was detected for *K* = 9, showing that the Bayesian analysis was able to distinguish more substructure in the dataset. At this level JO and MT appear to be different, whereas NG and UL were still not distinguished and admixed, especially with NQ. The admixture between these groups (NG, UL, NQ and DO) was further investigated in a substructure analysis (Figs 2B and S2B), where UL appeared as an admixture with NQ and NG populations.

**Fig 4:**
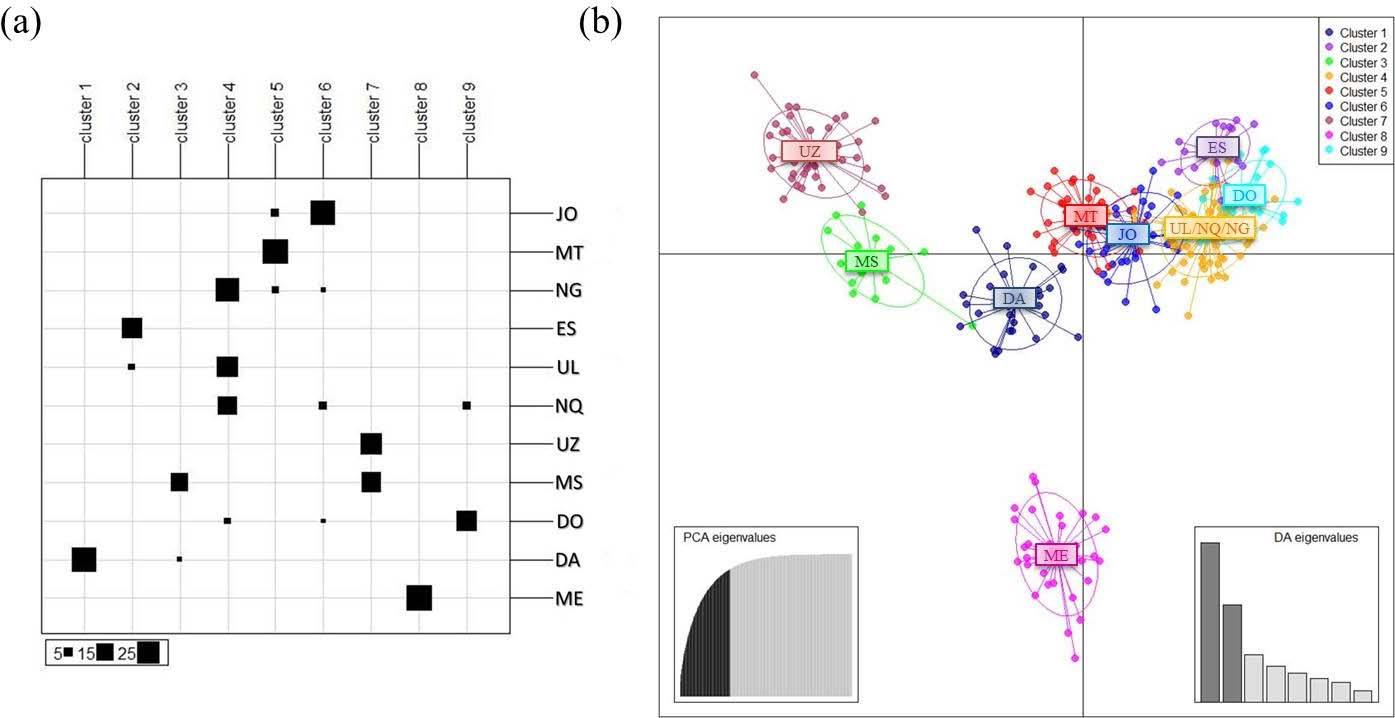
**Assignment of individuals to 9 clusters based on DAPC analyses (a). Scatterplot of the first two principal components of DAPC using populations as *a posteriori* clusters (b).** In Fig 4B, the individuals are assigned to populations *a posteriori*, that is, after determining the number of clusters by the programme, instead of forcing them into known populations. Populations are labeled inside their 95% inertia ellipses and dots represent individuals. The inset indicates the eigenvalues of the first four principal components and the variance explained by the PCA.

The Bayesian Information Criterion (BIC) statistic generated by Discriminant Analysis of Principal Components (DAPC) indicates that the optimal number of clusters in the data set is *K* = 9 (S2C Fig), showing five more clusters generated by DAPC than those generated by STRUCTURE. In the DAPC analysis, 80 PCs of the PCA were retained as input to discriminant analysis, accounting for approximately 89% of the total genetic variability. The scatterplot of the first two components of the DA (Fig 4A and 4B) showed extensive sharing of genetic variation among Zulu sheep. In particular, the plot showed that UZ and MS appeared clearly distinct from the other populations. The MT and JO populations showed genetic proximity and a particular affinity was observed among ES, UL, NQ, NG and DO. Using the grouping function obtained in the discriminant analysis, a high proportion of individuals were found to be correctly assigned to their original group: ES (100%), UZ (100%), MS (100%), MT (97%) UL (91%). The lowest scores were observed for JO (67%), NQ (64%) and NG (63%).

Moreover, the DAPC results are related to those of STRUCTURE at *K* = 9 presented in Fig 2.

Analysis of molecular variance (AMOVA) was performed to assess the variation within and between only Zulu populations. AMOVA revealed a high variance component within individuals (87.58%), followed by among populations (8.99%), and among individuals within populations (3.43%) (Table 3).

**Table 3:** Results from AMOVA analysis of only Zulu populations.

Source of variation	df	Sum of squares	Variance components	Percentage of variation	*F*-statistics
Among populations	7	67.147	0.15473 (Va)	8.99	*F*_ST_ = 0.08991*
Among individuals within populations	199	323.418	0.05899* (Vb)	3.43	*F*_IS_ = 0.03766
Within individuals	207	312.000	1.50725* (Vc)	87.58	*F*_IT_ = 0.12419*
**Total**	413	702.565	1.72097	100.00	

*P<0.01

The source of variation within populations (*Va*), among individuals within populations (*Vb*), within individuals (*Vc*) is given as a percentage for each comparison. *F*_IS_, genetic variation among groups; *F*_IT_, genetic variation among populations within groups; *F*_ST_, overall genetic variation among these populations.

## Discussion

A survey of Zulu sheep population size over a period of five years (2007 to 2011) revealed that population size decreased by 7.4% due to crossbreeding [16]. Consequently, critical attention should be paid to the population to stop their declining numbers. Official data on the census of the Zulu sheep is unavailable. The evaluation of genetic variation in Zulu sheep using a molecular approach is necessary for better understanding of the genetic diversity and structure of the sheep.

A total of 26 microsatellite loci revealed a recommended minimum number of alleles per locus [35]. The mean value of MNA of Zulu sheep (5.28) is similar to the one revealed by Buduram [36] in a study of South African sheep breeds (including Zulu sheep) assessed by 24 microsatellites loci. The mean values of H_O_ and H_E_ of the Zulu sheep were 0.58 and 0.63 respectively. In comparison, these values are closer to those revealed by Soma et al. [37] and Kunene et al. [20]. The values for H_O_ and H_E_ were above 0.50 in all the populations, indicating that the populations analysed are characterised by a noticeable genetic variation. The NQ had the highest values of observed and expected heterozygosity of all the populations which could be attributable to the large numbers of alleles detected. The lower mean number of alleles observed in ES is probably due to a recently reduced effective population size highlighted in the bottleneck analysis, because there were few farmers in this area who owned Zulu sheep. A bottleneck effect was also observed in UZ and MS, probably as a consequence of the small size of these two populations. The theory of genetics predicts that levels of genetic variation should increase with increasing effective population size, as bottlenecks entail genetic drift and inbreeding [38]. In comparison to other African indigenous sheep breeds, the genetic diversity indices (MNA, H_O_ and H_E_) of Zulu sheep were higher than Namaqua Afrikaner sheep [39]. Whereas, Nigerian indigenous sheep [40], Algerian indigenous sheep [41] and Egyptian indigenous sheep [42] had higher genetic diversity than the Zulu sheep.

The *F*_IS_ values for the majority of the populations were positive indicating some level of inbreeding. The JO and NG populations were relatively highly inbred compared to the rest of the Zulu sheep, followed by the NQ and UL. Similar findings were reported by Kunene et al. [20], where the authors found that some of the Zulu sheep populations were more inbred (Msinga) than others. Mavule et al. [16] reported that the Zulu sheep populations in 11 areas of KwaZulu Natal were genetically isolated; the authors revealed that 28% of the flocks did not interact with other flocks, but existed in isolation from neighbouring flocks and that at least 54% of the flocks interacted with 1 flock to a maximum of 3 flocks. Moreover, the majority of the farmers (66%) in these areas reported not practicing any form of inbreeding control [16]. Although the H_E_ and average number of alleles per locus were high in NQ, indicating a wide genetic base, the *F*_IS_ value indicate that the individuals in the population were inbred. The negative value (*F*_IS_ = -0.00642) for the sheep at ES was not significantly different from 0, indicating that rather than having excess heterozygotes, the population was not inbred. However, heterozygosity excess is usually developed when a population experiences a reduction of its effective size [43]. Although not significantly different from 0, the inbreeding coefficient (*F*_IS_) in the research stations (UZ, MS) was lower than that of the majority of the populations, indicating low inbreeding level. The *F*_IS_ values were lower than those reported by Kunene et al. [20], which were 0.0333 and 0.1178 for UZ and MS, respectively. The heterozygosity deficiency as a result of high level of inbreeding is not a threat only to Zulu sheep. The studies conducted on Moroccan [44] and Sudanese indigenous sheep [45] showed similar *F*_IS_ mean values to Zulu sheep, while studies conducted on Nigerian [46], Egyptian [47] and Tunisian indigenous sheep [48] had higher *F*_IS_ mean values than in Zulu sheep. Consequently, these results reveal that the level of inbreeding due to un-controlled mating strategies is a major problem in African local breeds.

The pair-wise *F*_ST_ values were computed to assess the level of genetic dilution in Zulu sheep due to crossbreeding with exotic sheep breeds. The lowest *F*_ST_ value (0.084) was found between the DO and NQ populations. Gaouar et al. [44] reported that the gene flow has an effective role in reducing the genetic differentiation among the breeds, particularly among those reared within the same or close geographical location. Among the Zulu sheep population, NQ had very low *F*_ST_ values, along with NG and UL (0.056 and 0.081, respectively). This could be the result of uncontrolled crossbreeding due to their geographical location. The phenomenon of crossbreeding has also been reported in Algerian breeds by Gaouar et al. [41] where Rembi and Taâdmit breeds were crossbred with Ouled-Djellal.

The Reynolds’ neighbor joining dendrogram showed that the populations ES, NG, UL and NQ had some considerable genetic influence from the Dorper breed. One of the purposes of the study was to investigate if there has been some introgression of the Zulu sheep with some of the exotic breeds. This was based on the survey conducted by Mavule et al. [16] where 43% of the farmers reported a history of crossbreeding. The farmers at UL and NQ specifically reported a history of genetic influence of Dorper and Merino on their Zulu sheep [16]. In addition, Mavule et al. [21] reported that the large size of the Nquthu sheep population is the result of crossbreeding with Dorper and Merino sheep. The Dorper is the second largest breed in South Africa. It is able to adapt to dry regions and produce mutton lambs in the harsh conditions in South Africa [49] [50]. The breed is larger in size than the Zulu sheep, which may have been one reason some farmers crossbred it with Zulu sheep to produce more meat. Nevertheless, the FAO [51] reported that almost 100 livestock breeds became extinct between 2000 and 2014, where country data revealed that the main cause of genetic erosion is crossbreeding.

A common genetic structure between the MT and JO population (*K* = 4) is probably caused by the geographic location. Zulu sheep farmers in these areas buy sheep from each other. Moreover, Mtubatuba is one of the areas with Zulu sheep distribution near the Jozini area [16]. The Zulu sheep populations at the two research stations (UZ and MS) formed one cluster and proved not to have any genetic introgression with any of the out-groups used in the study. The relationship between the populations in the research institutes was explained by Kunene et al. [20] as having some of the founder sheep purchased from common areas and controlled breeding management (exchange of breeding rams). Furthermore, the result of the structure assignment test confirmed the introgression of the Dorper with the four populations NG, ES, UL, and NQ (*K* = 4). The relationships between groups NG, UL, NQ and DO were further investigated (Fig 2B), with UL appearing as an admixture with the NG population. These two populations seem to have some of the genetic material observed in the NQ population. The study by Mavule et al. [21] showed that although NQ was larger in body measurements than the other Zulu sheep populations studied, due to crossbreeding, membership percentage of 11% of the population in NG could be classified as NQ using discriminant analysis. The current study may indicate that some of the genetic material found in NG could have been through the influence of crossbreeding. The close genetic relationship between the UL, NG and NQ may have been also caused by the effect of geographical location. The study by Mavule et al. [16] indicated that farmers got their founder flocks in some of the neighboring areas where the Zulu sheep were reported to be available.

As previously enunciated, the optimal number of clusters was revealed as *K* = 9, generated by Discriminant Analysis of Principal Component (DAPC). These clusters highlighted the uniqueness of each Zulu sheep population, which is a reflection of the large gene pool reported by Ramsay et al. [7]. Even the morphological studies have shown differences in traits such as coat colour, presence of horns, tail type within the same Zulu sheep populations in the different areas, leading the authors to conclude that individual populations could not be singled out as separate types [12] [22].

## Conclusions

Our study has shown that Zulu sheep are threatened by crossbreeding with exotic breeds, especially with the Dorper breed. Although some of the studied populations were affected by the admixture phenomenon there is still genetic diversity among populations. It can also be concluded from this work that Zulu sheep have some uniqueness among populations. Thus, there is a need for sustainable breeding and conservation programs to control the gene flow, in order to stop their possible genetic dilution.

## Materials and methods

### Ethics statement

All experimental procedures were reviewed and approved by the University of Zululand Research Ethics Committee, Reg No: UZREC 171110–030 PGM 2015/227.

### Animal sampling

A total of 207 blood samples of Zulu sheep were randomly collected from eShowe (ES), Makhathini research station (MS), Mtubatuba (MT), Jozini (JO), Nongoma (NG), Nquthu (NQ), Ulundi (UL) and UNIZULU research station (UZ) (S1 Fig). Data on the UZ and MS populations were obtained from a recent previous study that used the same microsatellite loci [20].

The eight populations used in the study were selected based on the availability of Zulu sheep in these areas. The details of the studied sheep populations are reported in S2 Table.

Blood samples were collected from each animal with the Vacutainer® system, in tubes with the addition of EDTA as anticoagulant, and stored at -20°C until analyses were performed. Analysed animals can be considered as a representative sample of the populations as they were chosen from different flocks, trying to avoid closely related individuals.

Due to probable crossbreeding of Nguni Zulu populations with exotic breeds which are common in the areas of KwaZulu Natal where the Zulu sheep are found; a total of 53 Dorper (DO) and South African Merino breed (ME) individuals were included in the dataset. In addition, 29 Damara (DA) animals were included as out-group.

### Molecular analysis

The GenElute Blood Genomic DNA kit (Sigma Aldrich, St. Louis, MO, USA) was used to extract the genomic DNA. Twenty-eight microsatellite loci (S1 Table) were selected from the list of recommended markers for genotyping analyses in sheep breeds [35]. The markers were selected based on degree of polymorphism and their position in the sheep genome. The microsatellite markers were optimised for multiplex PCR amplification using an ABI ProFlex PCR system under the same conditions reported in a previous work by Kunene et al. [20]. The multiplex PCR products were pooled to allow the analysis of more microsatellites in each electrophoresis. The size of the fragments was determined using an automated DNA sequencer (ABI 3500 Genetic Analyzer, Applied Biosystems, Foster City, CA, USA) and GeneMapper version 5.0 software (Applied Biosystems, Foster City, CA, USA).

### Statistical analysis

Allele frequencies, mean number of alleles, polymorphic information content (PIC) for each microsatellite loci, and the observed and expected heterozygosity in the eleven populations were estimated using the MICROSATELLITE TOOLKIT [52].

To calculate average allelic richness (R) and the richness of private alleles (PR) for each population, the rarefaction method [53] implemented in HP-RARE version 1.0 software was used, adopting a sample of 12 individuals [54]. A test for departure from Hardy-Weinberg equilibrium (HWE) was done using the Markov Chain Monte Carlo method (20 batches, 5,000 iterations per batch and a dememorisation number of 10,000) implemented in GENEPOP version 4.0 software [55]. Level of significance were adjusted using false discovery rate (FDR) procedure [56].

The *F*_IS_ for each population was calculated via bootstrapping using 1,000 replicates with GENETIX software version 4.05 [57]. The extent of population subdivision was investigated by calculating the global multi-locus *F*_ST_ value. The index of pairwise *F*_ST_ of Weir and Cockerham [58] between populations and their associated 95% confidence intervals was estimated using GDA software [59]. Bottleneck events in the Zulu populations were tested by the program BOTTLENECK version 1.2 [60] utilising the Wilcoxon test for heterozygote excess, as well as the two-phase model (TPM) recommended by Piry et al. [61] and Peery et al. [62].

The Reynolds’ weighted genetic distance [63] among the populations was calculated and a neighbour-joining tree was reconstructed using the PHYLIP package version 3.6 [64]; the dendrogram was depicted using the software package TreeView version 1.6.6 [65]. Bootstrap values were obtained with 1,000 replicates over the loci.

The hierarchical analysis of molecular variance (AMOVA) was performed using ARLEQUIN software version 3.5 [66] in order to quantify the degree of differentiation among populations.

Population structure across the entire dataset was analysed using a Bayesian approach implemented in STRUCTURE software version 2.3.4 [67] to assess the most probable number of partitions in the dataset without the assumption of the breed identities. The assignment of individuals to populations considered an ancestry model with admixture, correlated allele frequencies, and defined sampling location for each individual. Ten independent runs with 500,000 MCMC (Markov Chain Monte Carlo) iterations and a burn-in of 200,000 steps were performed for 2 ≤ *K* ≤ 13 (*K* = number of clusters) to estimate the most likely number of clusters present in the dataset. The algorithm of Evanno et al. [68] was adopted in order to evaluate the most probable value of *K*. Moreover, in order to investigate population substructures, the most interesting cluster identified with STRUCTURE was re-analysed using the same settings and assuming *K* = 2 to *K* = n+3 (n being the number of populations included in each cluster). STRUCTURE HARVESTER [69], a web-based program, was used for collating the results generated by the program STRUCTURE. The clustering pattern was implemented in the CLUMPP program and visualised using the software DISTRUCT software version 1.1 [70].

To further investigate the genetic structure of each population when adopting an approach without assumptions about HWE or linkage disequilibrium, Discriminant Analysis of Principal Component (DAPC) was carried out with the method implemented in the ADEGENET package [71] within the statistical package R version 3.3.2 [72]. DAPC was conducted without *a posteriori* group assignments by inferring the most likely number of genetic clusters (*K*) using the *find*.*clusters* function in the ADEGENET package. This function utilises *K*-means clustering to calculate a Bayesian information criterion (BIC) value for each potential value of *K* (the most likely *K* has the lowest BIC value) and delineates individual group assignments for DAPC.

## Supporting information

S1 Table Microsatellite loci, chromosomal position (Chr), size range (S.R.), genebank accession number and references, number of alleles observed (Na) at each locus, expected (H_E_) and observed (H_O_) heterozygosity at each locus, mean PIC (polymorphic information content) per locus in the eleven studied sheep populations and number of populations deviated from the Hardy-Weinberg equilibrium per locus (HWE Pop). (PDF)

S2 Table Details for the eleven South African sheep breeds/populations; phenotypic description, geographic localization, adaptive traits, and management practices. (PDF)

S3 Table Data file of microsatellite allele lengths for the 28 loci utilised in the paper. The data file has individuals organised in rows, with name of population in the first column. The subsequent columns correspond to microsatellite data (two columns per locus). Missing data are coded by zero.
(CSV)

S1 Fig Geographical location of the sampling sites for the studied South African populations. JO, Jozini: latitude: 27° 42' 94"S longitude: 32° 06' 57"E; MT, Mtubatuba latitude: 28° 40' 59"S longitude: 32° 21' 43"E; NG, Nongoma latitude: 27° 89' 43"S longitude: 31° 64' 54"E; ES, Eshowe latitude: 28° 89' 47"S longitude: 31° 46' 28"E; UL, Ulundi latitude: 28° 29' 97"S longitude: 31° 43' 42"E; NQ, Nquthu latitude: 28° 30' 08"S longitude: 30° 80' 39"E; UZ, UNIZULU research station latitude: 28° 85' 24"S longitude: 31° 84' 91"E; MS, Makhathini research station latitude: 27° 39' 53"S longitude: 32° 17' 64"E; DO, Dorper latitude: 29° 80' 00"S longitude: 30° 65' 00"E; DA, Damara latitude: 25° 16' 74"S longitude: 29° 39' 87"E; ME, South African Merino latitude: 29° 60' 06"S longitude: 30° 37' 94"E and latitude: 29° 98' 25'' S longitude:30° 92' 17''ESource: figure taken from http://www.d-maps.com and adapted for illustrative purpose only.
(TIF)

S2 Fig Delta *K* plots of STRUCTURE analysis averaged over ten repetitions at *K* = 1 to 13 (a). Bayesian information criterion (BIC) values plotted for number of clusters ranging from *K* = 1 to 40 derived from Discriminant Analysis of Principal Components (DAPC) (b). Delta *K* distribution of sub-STRUCTURE analysis (c) Delta *K* distribution of sub-STRUCTURE analysis obtained from NG, UL, NQ and DO.
(TIF)
