# Supplementary material for: Genetic structure of South African Nguni (Zulu) sheep populations reveals admixture with exotic breeds
Source: PLoS One. 2018 Apr 26;13(4):e0196276. doi: 10.1371/journal.pone.0196276 (PMC5919407; doi:10.1371/journal.pone.0196276)
Supplement: S1 Table — (PDF) [file pone.0196276.s001.pdf]

Locus	Chr.	S.R. (bp)	Genebank accession number	References	Na	H_E	H_O	PIC	HWE Pop†
BM1824	1	169-177	G18394	[1]	7	0.66	0.65	0.59	3
BM6506	1	192-208	G18455	[1]	9	0.56	0.57	0.51	0
INRA006	1	101-141	X637951	[2]	12	0.60	0.59	0.53	1
OarFCB11	2	117-151	L01531	[3]	11	0.77	0.75	0.72	0
OarFCB20	2	106-130	L20004	[4]	14	0.78	0.78	0.72	1
D5S2*	5	166-192	Z22743	[5]	8	0.45	0.42	0.50	0
ETH10*	5	190-206	Z22739	[5]	3	0.13	0.09	0.11	1
RM006	5	205-211	-	[6]	11	0.67	0.63	0.60	0
McM527	5	113-141	L34277	[7]	13	0.75	0.71	0.69	1
ETH225	9	134-156	-	[8]	13	0.52	0.38	0.51	4
CSSM66	9	165-225	-	[9]	18	0.57	0.55	0.53	1
ILSTS11	9	201-288	L23485	[10]	10	0.48	0.42	0.50	2
INRA035	12	112-138	X68049	[2]	11	0.71	0.60	0.65	0
TGLA53	12	147-167	-	[11]	13	0.79	0.78	0.75	2
CSRD247*	14	213-259	EU009450	[12]	13	0.66	0.58	0.61	2
INRA063	14	157-195	X71507	[2]	12	0.60	0.49	0.54	2
SPS115	15	234-250	-	[13]	10	0.56	0.48	0.51	0
MAF65	15	128-144	M67437	[14]	10	0.72	0.78	0.66	3
TGLA126	16	119-147	-	[15]	17	0.77	0.44	0.72	8
MAF214	16	107-137	M88160	[16]	10	0.49	0.49	0.50	0
MAF209	17	108-140	M80358	[17]	13	0.74	0.64	0.68	1
BM8125	17	141-171	G18475	[1]	8	0.51	0.48	0.50	0
OarFCB48	17	138-174	M82875	[4]	10	0.45	0.33	0.51	4
OarFCB304	19	151-194	L01535	[3]	14	0.75	0.73	0.70	0
HSC	20	268-300	M90759	[18]	19	0.84	0.70	0.80	4
BM1818	20	256-284	G18391	[1]	14	0.73	0.72	0.67	2
OarCP20	21	68-94	-	[19]	9	0.61	0.52	0.52	0
BM6526	26	151-175	-	[1]	11	0.69	0.58	0.63	2
					11.54	0.63	0.57	0.59	

*: relative to cattle linkage map (not mapped in *Ovis aries*).

†: after Benjamini and Hochberg [20] correction

In grey, the markers excluded from the further analysis on the bases of PIC values and HWE equilibrium

1. Bishop MD, Kappes SM, Keele JW, Stone RT, Sunden SL, Hawkins SL, et al. A genetic linkage map for cattle. Genetics. 1994; 136: 619–639.
2. Vaiman D, Mercier D, Moazami–Goudarzi K, Eggen A, Ciampolini R, Lépingle A, et al. A set of 99 cattle microsatellites: characterization, synteny mapping, and polymorphism. Mamm Genome. 1994; 5: 288–297.
3. Buchanan FC, Crawford AM. Ovine microsatellites at the OarFCB11, OarFCB128, OarFCB193, OarFCB266 and OarFCB304 loci. Anim Genet. 1993; 24: 145.
4. Buchanan FC, Galloway SM, Crawford AM. Ovine microsatellites at the OarFCB5, OarFCB19, OarFCB20, OarFCB48, OarFCB129 and OarFCB226 loci. Anim Genet. 1994; 25: 60.
5. Toldo SS, Fries R, Steffen P, Neibergs HL, Barendse W, Womack JE, Hetzel DJ, Stranzinger G. Physically mapped, cosmid-derived microsatellite markers as anchor loci on bovine chromosomes. Mamm Genome. 1993;4 :720–727.
6. Kossarek LM, Grosse WM, Finlay O, McGraw RA. Rapid communication: bovine dinucleotide repeat polymorphism RM006. J Anim Sci. 1993; 71: 3176–3176.
7. Hulme DJ, Silk JP, Redwin JM, Barendse W, Beh KJ. Ten polymorphic ovine microsatellites. Anim Genet. 1994; 25: 434–435.
8. Steffen P, Eggen A. Isolation and mapping of polymorphic microsatellites in cattle. Anim. Genet. 1993; 24: 121–124.
9. Arora R, Lakhchaura BD, Prosad RB, Chauhan A, Bais RKS, Tantia MS, Vijh RK. Physical and microsatellite based characterization of Tarai Buffalo and of India Buffalo. Newsletter. 2003; 19.
10. Brezinsky L, Kemp SJ, Teale AJ. Five polymorphic bovine microsatellites (ILSTS010–014). Anim Genet. 1993; 24: 75–76.

11. Georges M, Massey J. Polymorphic DNA markers in Bovidae (World Intellectual Property Org Geneva). WO Publ 92/13102; 1992.

12. Davies KP, Maddox JF, Harrison B, Drinkwater R. Ovine dinucleotide repeat polymorphism at eight anonymous loci. Anim Genet. 1996; 27: 381–382.

13. Baylor College of Medicine Human Genome Sequencing Center. Bovine Whole Genome Assembly release Btau_3.1. 2006. Available from: https://www.hgsc.bcm.edu/other-mammals/bovine-genome-project.

14. Buchanan FC, Swarbrick PA, Crawford AM. Ovine dinucleotide repeat polymorphism at the MAF65 locus. Anim Genet. 1992; 23: 85.

15. Kappes SM, Keele JW, Stone RT, McGraw RA, Sonstegard TS, Smith TP, Lopez-Corrales NL, Beattie CW. A second generation linkage map of the bovine genome. Genome Res. 1997; 7: 235–249.

16. Buchanan FC, Crawford AM. Ovine dinucleotide repeat polymorphism at the MAF214 locus. Anim Genet. 1992; 23: 394.

17. Buchanan FC, Crawford AM. Ovine dinucleotide repeat polymorphism at the MAF209 locus. Anim Genet. 1992; 23: 183.

18. Scott PC, Maddox JF, Gogolin–Ewens KJ, Brandon MR. The nucleotide sequence and evolution of ovine MHC class II B genes: DQB and DRB. Immunogenetics. 1992; 35: 217.

19. Ede AJ, Pierson CA, Crawford AM. Ovine microsatellites at the OarCP9, OarCP16, OarCP20, OarCP21, OarCP23 and OarCP26 loci. Anim Genet. 1995; 26: 129–130.

20. Benjamini Y, Hochberg Y. Controlling the false discovery rate: a practical and powerful approach to multiple testing. J R Stat Soc Series B Stat Methodol. 1995; 289-300.
