# Supplementary material for: Genetic structure of South African Nguni (Zulu) sheep populations reveals admixture with exotic breeds
Source: PLoS One. 2018 Apr 26;13(4):e0196276. doi: 10.1371/journal.pone.0196276 (PMC5919407; doi:10.1371/journal.pone.0196276)
Supplement: S2 Table — (PDF) [file pone.0196276.s002.pdf]

Population /breed ǂ	Phenotypic description	Geographic localization	Adaptive traits	Management practices
**JO**	Fat-tailed hair sheep. Coat colour: dark-brown and white, dark-brown, black and white. Horn absent.	latitude: 27° 42' 94"S longitude: 32° 06' 57"E	Hot and wind (adapted to long walks)	Extensive sheep production system
**MT**	Long fat-tailed Hair sheep. Coat colour: dark-brown, brown, fawn, dark-brown and white, brown and white, fawn and white, black and white. Horn absent.	latitude: 28° 40’ 59’’S longitude: 32° 21’ 43’’E	Hot and cold (adapted to long walks)	Extensive sheep production system
**NG**	Long fat-tailed hair sheep. Coat colour: dark-brown, brown, fawn, dark-brown and white, brown and white, fawn and white, black and white. Horn absent.	latitude: 27° 89’ 43’’S longitude: 31° 64’ 54’’E	Hot and cold (adapted to long walks)	Extensive and semi-intensive sheep production system
**ES**	Long fat-tailed hair sheep. Coat colour: dark-brown, brown, fawn. Horn absent.	latitude: 28° 89’ 47’’S longitude: 31° 46’ 28’’ E	Hot and cold (adapted to long walks)	Extensive sheep production system
**UL**	Long fat-tailed hair sheep. Coat colour: dark-brown, fawn, dark-brown and white, brown and white, black and white. Horn absent.	latitude: 28° 29’ 97’’S longitude: 31° 43’ 42’’E	Hot and cold (adapted to long walks)	Extensive sheep production system
**NQ**	Long fat-tailed hair sheep. Coat colour: dark-brown, fawn, dark-brown and white, brown and white, black, black and white. Horn absent.	latitude: 28° 30’ 08’’S longitude: 30° 80’ 39’’E	Hot and cold (adapted to long walks)	Extensive sheep production system

**UZ**	Long fat-tailed and thin tailed hair sheep.<br>Coat colour: dark-brown, brown, fawn, dark-brown and white, brown and white, black, black and white.<br>Horn absent.	Pilot farm<br>latitude: 28° 85’ 24’’S longitude: 31° 84’ 91’’E	Hot and cold	Semi-intensive sheep production system
**MS**	Long fat-tailed and thin-tailed hair sheep<br>Coat colour: dark-brown, brown, fawn, dark-brown and white, brown and white, black, black and white.<br>Horn absent.	Pilot farm<br>latitude: 27° 39’ 53’’S longitude: 32° 17’ 64’’E	Hot and cold	Semi-intensive sheep production farming
**DO**	Short tailed sheep.<br>Coat colour: black and white<br>Horn absent.	latitude: 29° 80’ 0’’S longitude: 30° 65’ 0’’E	Arid to semi-arid conditions	Semi-intensive sheep production system
**DA**	Long fat tailed hair sheep.<br>Coat colour: black and white, brown and white.<br>Horn present.	latitude: 25° 16’ 74’’S longitude: 29° 39’ 87’’E	Arid, semi-desert areas	Semi-intensive sheep production system
**ME**	Wool sheep.<br>Coat colour: white<br>Horn absent.	Site 1: Pietermaritzburg, latitude: 29° 60' 06"S longitude: 30° 37' 94"E<br>Site 2: Isipingo, latitude: 29° 98' 25" S longitude:30° 92' 17"E	Semi-arid areas up to high rainfall regions	Semi-intensive sheep production system

ǂ Official data on census of the Zulu sheep is unavailable.

JO, Jozini; MT, Mtubatuba; NG, Nongoma; ES, Eshowe; UL, Ulundi; NQ, Nquthu; UZ, UNIZULU research station; MS, Makhathini research station; DO, Dorper; DA, Damara; ME, South African Merino.
