# Supplementary figures and images for: Genetic structure of South African Nguni (Zulu) sheep populations reveals admixture with exotic breeds
Source: PLoS One. 2018 Apr 26;13(4):e0196276. doi: 10.1371/journal.pone.0196276 (PMC5919407; doi:10.1371/journal.pone.0196276)

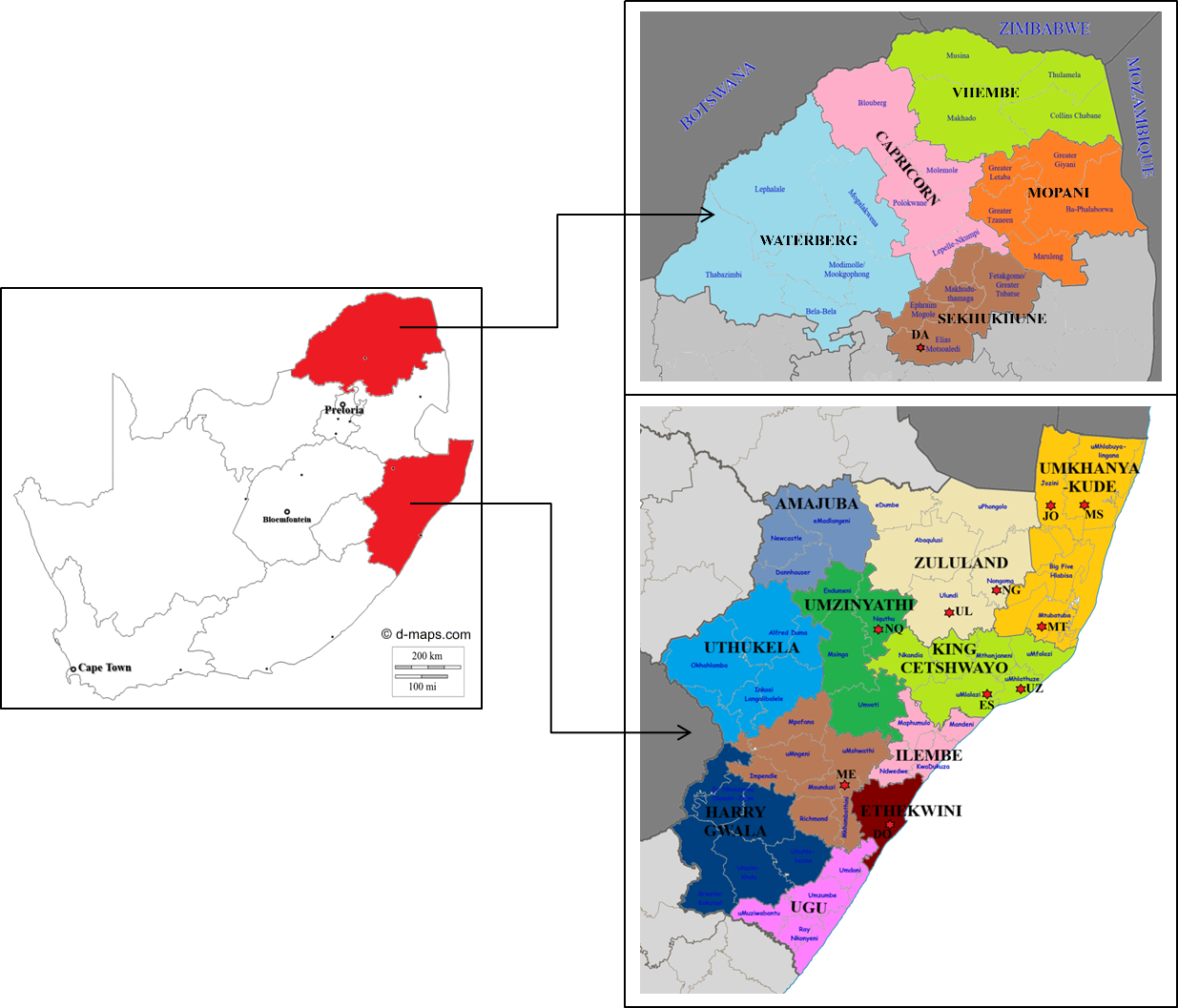

Supplement: S1 Fig — JO, Jozini: latitude: 27° 42' 94"S longitude: 32° 06' 57"E; MT, Mtubatuba latitude: 28° 40' 59"S longitude: 32° 21' 43"E; NG, Nongoma latitude: 27° 89' 43"S longitude: 31° 64' 54"E; ES, Eshowe latitude: 28° 89' 47"S longitude: 31° 46' 28"E; UL, Ulundi latitude: 28° 29' 97"S longitude: 31° 43' 42"E; NQ, Nquthu latitude: 28° 30' 08"S longitude: 30° 80' 39"E; UZ, UNIZULU research station latitude: 28° 85' 24"S longitude: 31° 84' 91"E; MS, Makhathini research station latitude: 27° 39' 53"S longitude: 32° 17' 64"E; DO, Dorper latitude: 29° 80' 00"S longitude: 30° 65' 00"E; DA, Damara latitude: 25° 16' 74"S longitude: 29° 39' 87"E; ME, South African Merino latitude: 29° 60' 06"S longitude: 30° 37' 94"E and latitude: 29° 98' 25'' S longitude:30° 92' 17''ESource: figure taken from http://www.d-maps.com and adapted for illustrative purpose only. (TIF) [file pone.0196276.s004.tif]

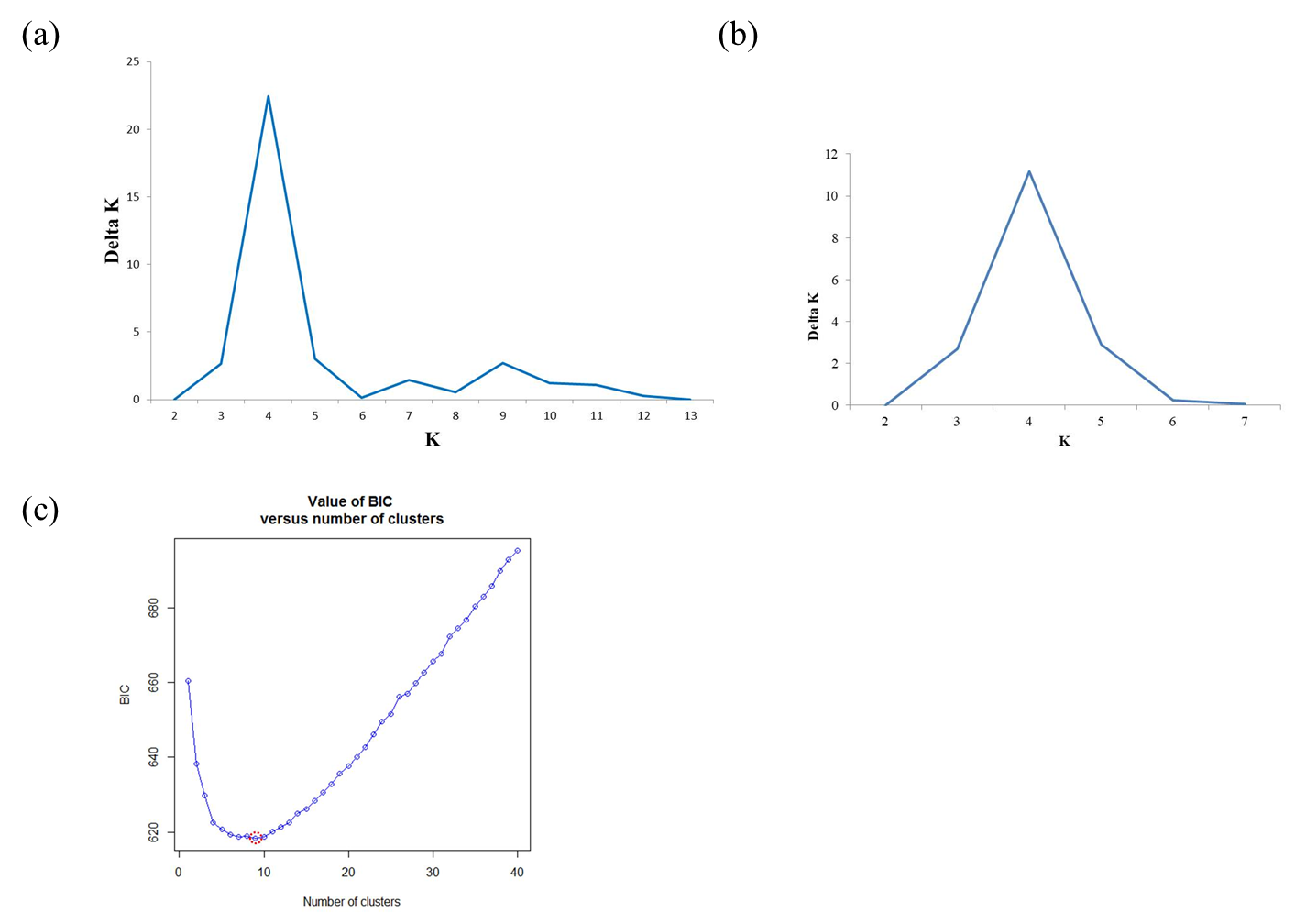

Supplement: S2 Fig — Delta K plots of STRUCTURE analysis averaged over ten repetitions at K = 1 to 13 (a). Bayesian information criterion (BIC) values plotted for number of clusters ranging from K = 1 to 40 derived from Discriminant Analysis of Principal Components (DAPC) (b). Delta K distribution of sub-STRUCTURE analysis (c) Delta K distribution of sub-STRUCTURE analysis obtained from NG, UL, NQ and DO. (TIF) [file pone.0196276.s005.tif]
